# Among the Colleges

**Published:** 1898-11

**Authors:** 


### AMONG THE COLLEGES.

The three graduates of the Western Veterinary College, located at Kansas City, Mo., were former students in the Ontario, Ohio Veterinary College (Cincinnati), and Kansas City Veterinary College.

Two free scholarships to each county in the State are allotted by the Ohio State University, Veterinary Department.

The Kansas City Veterinary College conducts a ladies' class in microscopy preparatory to the civil-service examinations for assistants in the meat-inspection department of the Bureau of Animal Industry.

The Grand Rapids Veterinary College opened with some ten students on October 11th, one of whom was a lady. The prospects for an additional number of students is quite promising. The school has planned to give special instruction in clinical work during the session, and a large amount of material has been engaged for surgical instruction in the various operations upon cryptorchids and other animals. The free clinics for the poor are reported as being taken advantage of to an unusual extent.

